# Research advances in the application of microfluidic chip technology for rapid detection of antibiotic-resistant bacteria

**DOI:** 10.3389/fcimb.2026.1819856

**Published:** 2026-05-11

**Authors:** Lijun Xia, Changqing Tang, Dalong Tong, Qifu He

**Affiliations:** 1 Wuhou District People’s Hospital, Chengdu, China; 2 Wuhou District Health Hospital Woman and Children, Chengdu, China

**Keywords:** antibiotic-resistant bacteria, antimicrobial susceptibility testing, microfluidic chip, point-of-care diagnostics, rapid detection

## Abstract

The escalating global burden of antimicrobial resistance (AMR) necessitates diagnostic strategies that can overcome the limitations of conventional culture-based methods, which often require several days to generate clinically actionable results. Such delays are associated with increased mortality, inappropriate antibiotic use, and continued transmission of resistant pathogens. In this context, microfluidic chip technology has emerged as a promising platform for rapid, miniaturized, and increasingly automated point-of-care diagnostics. Recent advances have enabled integrated lab-on-a-chip systems that combine bacterial isolation, phenotypic antimicrobial susceptibility testing, and genotypic resistance detection within closed and self-contained architectures, thereby reducing contamination risk and operator dependence. In addition, these platforms are increasingly capable of operating at single-cell resolution, allowing the detection of heteroresistance and resistant subpopulations that may be overlooked by conventional bulk assays. A major advantage of microfluidic systems is their ability to bridge phenotypic and genotypic diagnostics by enabling real-time monitoring of bacterial growth, metabolic activity, and morphological responses to antibiotics while simultaneously incorporating on-chip nucleic acid amplification for resistance gene detection. This integrated approach improves the interpretation of discrepancies between genetic determinants and functional resistance. Studies to date have demonstrated high sensitivity and specificity in complex clinical matrices, including blood, urine, and sputum, with turnaround times reduced from days to less than one hour in some applications. Furthermore, the integration of CRISPR-Cas systems, nanomaterial-enhanced biosensing, and machine learning has further improved analytical performance and data interpretation. Nevertheless, important translational challenges remain, including scalable manufacturing, regulatory standardization, and integration into routine clinical workflows. Future microfluidic platforms are expected to support multiplexed, intelligent antimicrobial susceptibility testing capable of simultaneous pathogen identification, resistance profiling, and therapeutic guidance, thereby advancing precision diagnostics for AMR management.

## 1 Introduction

Antimicrobial resistance (AMR) poses a critical and escalating threat to global public health. In 2023 alone, approximately one in every six laboratory-confirmed bacterial infections globally was resistant to antibiotic treatment, and this resistance is not static; between 2018 and 2023, over 40% of monitored pathogen-antibiotic combinations saw rising resistance rates. This relentless progression directly translates into grave clinical outcomes, where delayed detection strongly contributes to increased mortality, prolonged hospital stays, and the inappropriate use of broad-spectrum antibiotics (Wang et al., 2025). For instance, a 2024 patient-level analysis attributed 4.2% of all deaths in a UK hospital directly to AMR, highlighting its significant fatal impact. Conventional culture-based diagnostics still considered the clinical gold standard—require 24–72 hours for antimicrobial susceptibility testing (AST), creating a dangerous lag in effective therapeutic decision-making, particularly in sepsis and other acute infections (Wistrand-Yuen et al., 2020). This diagnostic gap is especially detrimental in critical care and low-resource settings where access to advanced microbiology infrastructure is scarce, yet the burden of resistant pathogens, including those prioritized by the World Health Organization (WHO), remains high (Joh et al., 2023). In these settings, infections with multidrug-resistant pathogens significantly extend Intensive Care Unit (ICU) stays and elevate mortality risks. In response, microfluidic chip technology has emerged as a transformative platform for rapid, point-of-care detection of antibiotic-resistant bacteria, offering sample-to-answer workflows that compress turnaround times from days to hours or even minutes (Hsieh et al., 2021; Song et al., 2022).

Recent advances in microfluidics have enabled integrated lab-on-a-chip systems. These compact platforms are increasingly capable of performing both phenotypic and genotypic resistance profiling with high sensitivity within streamlined workflows that require minimal hands-on time (Lee et al., 2020). A key strength lies in their ability to support real-time monitoring of bacterial responses to antibiotics at the single-cell level. This capability is particularly important because it can reveal resistant subpopulations and phenotypic heterogeneity that are often overlooked by conventional bulk assays (So, 2023). In parallel, on-chip nucleic acid amplification strategies, encompassing both isothermal and PCR-based approaches, now allow us to directly pinpoint specific resistance genes from challenging clinical samples like blood, urine, and sputum (Fang et al., 2020; Li et al., 2023). By converging these two diagnostic modalities, the technology tackles a persistent clinical puzzle: bridging the sometimes frustrating gap between the presence of a genetic marker and the actual expression of functional resistance. This integration is vital for generating accurate, actionable data to guide therapy (So, 2023).

**Figure 1:**
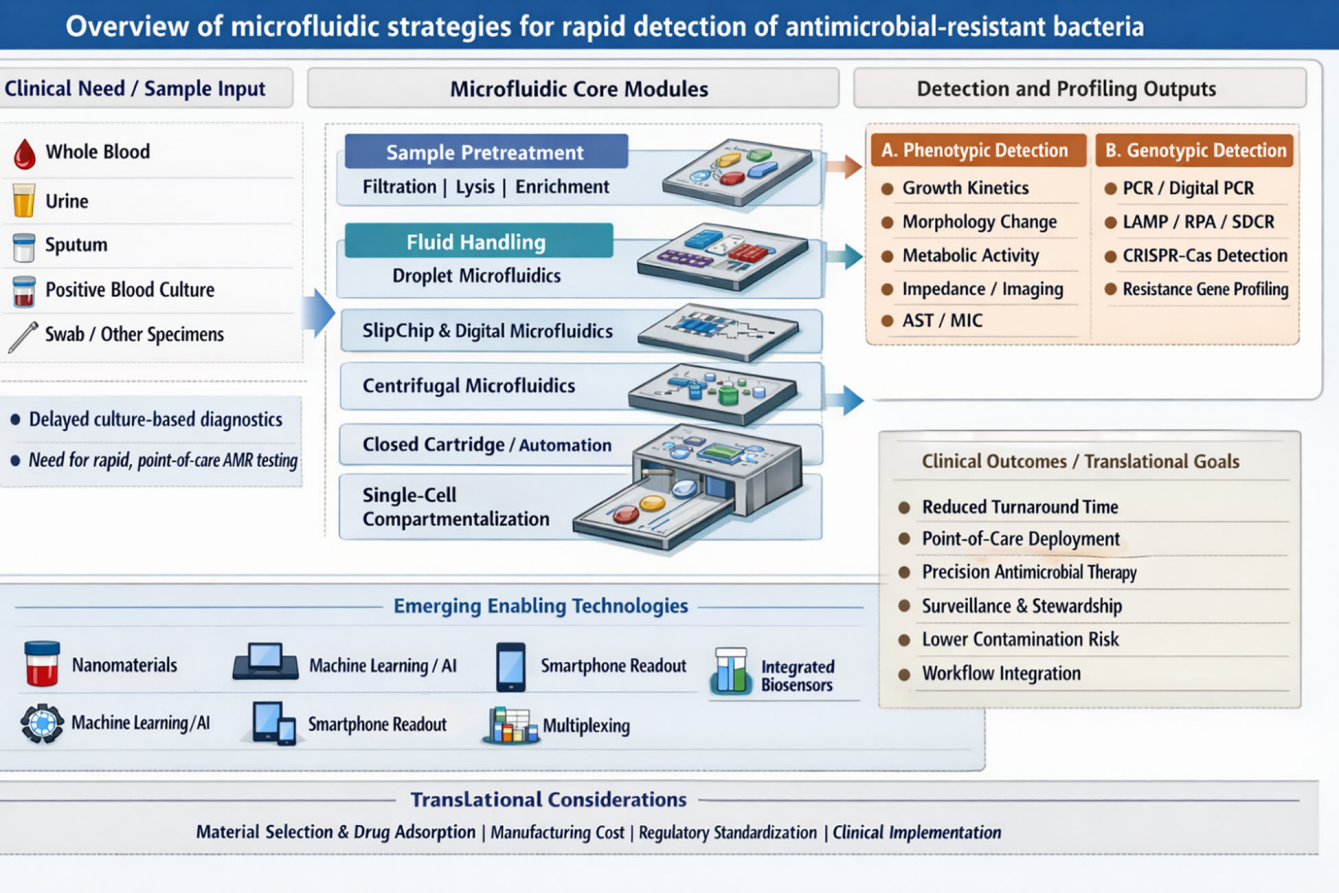
Overview of microfluidic strategies for rapid detection of antimicrobial-resistant bacteria.

Moreover, the true potential of these microfluidic platforms is being unlocked by integrating them with other cutting-edge technologies. CRISPR-Cas technology, derived from the adaptive immune systems of bacteria and archaea, uses guide RNA-directed Cas nucleases to recognize specific nucleic acid sequences with high specificity. In diagnostic applications, target recognition by enzymes such as Cas12 or Cas13 can trigger collateral cleavage of labeled reporter molecules, thereby converting sequence recognition into a measurable fluorescent, colorimetric, or electrochemical signal. When integrated with microfluidic chips, these reactions can be performed in closed, miniaturized systems that support rapid and point-of-care detection of resistance determinants (Tong et al., 2022; Ma et al., 2023). Despite these innovations, translational barriers persist, including manufacturing costs, regulatory approval pathways, and seamless integration into diverse clinical workflows—from intensive care units to community clinics (Duarte et al., 2024). This review, therefore, aims to connect these dots. We synthesize the current state of microfluidic chip technology for fighting antibiotic-resistant bacteria, critically evaluate its performance against the gold standards of clinical practice, and chart a course for the future. The ultimate goal is the development of intelligent, multiplexed diagnostic systems. Such platforms could fundamentally reshape antimicrobial management and improve patient outcomes worldwide. To provide a conceptual framework for the discussion that follows, **Figure 1** summarizes the major roles of microfluidic technologies in AMR diagnostics, including sample preparation, single-cell isolation, phenotypic antimicrobial susceptibility testing, on-chip nucleic acid amplification, CRISPR-based detection, nanomaterial-enhanced signal transduction, and machine learning-assisted data interpretation.

## 2 Clinical imperatives driving the need for rapid diagnostics in antimicrobial resistance

### 2.1 Limitations of conventional culture-based methods in critical care settings

Conventional culture-based methods remain the historical cornerstone of microbial identification and AST, yet their inherent limitations are increasingly incompatible with the demands of modern critical care. Standard blood culture protocols typically require 24–72 hours for preliminary pathogen identification and an additional 24–48 hours for full AST results, creating a diagnostic void during which clinicians must rely on empirical broad-spectrum antibiotic regimens (Shanmugakani et al., 2020; Eubank et al., 2021). In intensive care units (ICUs), where sepsis and multidrug-resistant (MDR) infections progress rapidly, this delay significantly compromises therapeutic precision. Empirical therapy, while life-saving in acute settings, often leads to overuse of last-resort antibiotics such as carbapenems or colistin, accelerating resistance development and increasing the risk of collateral damage to the patient’s microbiome (Spadaro, 2023; Pickens and Wunderink, 2024).

Moreover, culture-based techniques suffer from suboptimal sensitivity, particularly in patients who have already received antimicrobial therapy prior to sample collection—a common scenario in tertiary hospitals. Studies indicate that pre-antibiotic administration can reduce culture positivity by up to 50%, thereby obscuring the true etiology of infection (Rentschler et al., 2021; Liborio et al., 2024). Additionally, fastidious organisms such as *Haemophilus influenzae*, anaerobes, and certain fungi may not grow under standard culture conditions, leading to false-negative results and missed opportunities for targeted intervention (Lass-Flörl, 2024). This diagnostic gap is especially pronounced in immunocompromised patients, where polymicrobial infections or low-burden pathogens evade culture detection but drive significant morbidity. The inability of culture methods to detect resistance mechanisms at the genetic level—such as extended-spectrum β-lactamase (ESBL) or carbapenemase genes—further limits their utility in guiding early de-escalation or escalation strategies (Shanmugakani et al., 2020). Consequently, the reliance on culture in critical care not only delays appropriate therapy but also impedes effective antimicrobial stewardship programs aimed at curbing resistance propagation.

### 2.2 Global health burden of delayed resistance detection

The clinical consequences of delayed antimicrobial resistance detection extend far beyond individual patient outcomes, contributing to a measurable global health burden characterized by excess mortality, prolonged hospitalization, and unsustainable antibiotic consumption (Gopal et al., 2021). A landmark meta-analysis of sepsis management demonstrated that each hour of delay in administering appropriate antimicrobial therapy increases mortality by 7.6% (Eubank et al., 2021). In the context of MDR pathogens—such as carbapenem-resistant Enterobacteriaceae (CRE) or methicillin-resistant *Staphylococcus aureus* (MRSA)—this translates into thousands of preventable deaths annually (Li et al., 2023). The WHO estimates that AMR already accounts for approximately 1.27 million direct deaths globally per year, a figure projected to rise to 10 million by 2050 if current trends persist (Udaondo and Matilla, 2020; Eisinger et al., 2023).

Delayed diagnostics directly fuel antibiotic misuse. Empirical regimens often involve unnecessarily broad coverage, including combinations of β-lactams, aminoglycosides, and glycopeptides, even when narrower-spectrum agents would suffice. A 2022 ICU study revealed that 68% of initial antibiotic prescriptions were discordant with final culture-based susceptibility results, with 41% representing overtreatment (Spadaro, 2023). This practice not only selects for resistant clones within hospitals but also disrupts ecological balances in community microbiomes, facilitating horizontal gene transfer of resistance determinants (Merker et al., 2020). Furthermore, prolonged use of broad-spectrum agents increases the risk of Clostridioides difficile infection and invasive candidiasis, compounding morbidity and healthcare costs (Pickens and Wunderink, 2024).

Economic analyses reinforce the urgency. In the United States alone, AMR-related hospitalizations cost an estimated $20 billion annually in excess expenditures, with diagnostic delays accounting for nearly 30% of this burden due to extended ICU stays and readmissions (Liborio et al., 2024). Similarly, deployment of rapid carbapenemase detection assays in European ICUs led to a 25% reduction in carbapenem consumption within six months, demonstrating tangible stewardship benefits (Martínez Sagasti et al., 2022). Critically, the impact of turnaround time is nonlinear—small reductions in diagnostic latency yield disproportionately large gains in survival when applied during the “golden hour” of sepsis management (Blondeau and Rankin, 2021). Thus, the global health imperative is not merely to develop faster tests but to integrate them into clinical workflows where they can alter decision-making in real time. Without such integration, even the most advanced diagnostic remains academically impressive but clinically inert.

### 2.3 WHO priority pathogens and the urgency for point-of-care solutions in resource-stratified healthcare systems

The WHO Bacterial Priority Pathogens List (BPPL) 2024 has clearly prioritized antibiotic-resistant bacteria based on factors such as global severity, transmission ability, treatment feasibility, prevention possibility, and resistance trends. In the updated WHO BPPL, the critical-priority tier includes carbapenem-resistant *Acinetobacter baumannii*, third-generation cephalosporin-resistant Enterobacterales, carbapenem-resistant Enterobacterales, and rifampicin-resistant *Mycobacterium tuberculosis*. The high-priority tier includes vancomycin-resistant *Enterococcus faecium*, carbapenem-resistant *Pseudomonas aeruginosa*, methicillin-resistant *Staphylococcus aureus*, fluoroquinolone-resistant *Salmonella Typhi*, non-typhoidal *Salmonella*, fluoroquinolone-resistant *Shigella* spp., and cephalosporin- and/or fluoroquinolone-resistant *Neisseria gonorrhoeae* (Sati et al., 2025; Wise, 2025). These pathogens merit particular diagnostic attention because they combine high clinical burden with limited treatment options, strong transmission potential in healthcare or community settings, or both.

Regional surveillance data further explain why these pathogens should remain central to rapid diagnostic development. According to the WHO GLASS 2025 analysis, one in six laboratory-confirmed bacterial infections worldwide in 2023 was resistant to antibiotic treatment, with the highest resistance burden observed in the WHO South-East Asian and Eastern Mediterranean Regions, where approximately one in three reported infections were resistant; in the African Region, one in five infections was resistant. The same WHO update noted that more than 40% of *Escherichia coli* and more than 55% of *Klebsiella pneumoniae* globally are now resistant to third-generation cephalosporins, with resistance exceeding 70% in the African Region (Jesudason, 2024). In Europe, the estimated incidence of carbapenem-resistant Klebsiella pneumoniae bloodstream infection reached 3.51 per 100–000 population in 2024, representing a 61.0% increase compared with 2019, while MRSA bloodstream infection incidence remained 4.48 per 100–000 and vancomycin-resistant *E. faecium* bloodstream infection incidence reached 1.96 per 100–000 (Lowe et al., 2022; Graham et al., 2024). In the United States, CDC reported that six bacterial hospital-onset antimicrobial-resistant infections increased by a combined 20% during the COVID-19 period and, by 2022, all but MRSA remained above pre-pandemic levels (Cartwright et al., 2024; Kuehn et al., 2025).

Outbreak potential and diagnostic access further justify prioritization. ECDC’s 2025 risk assessment concluded that the probability of further spread of carbapenem-resistant Enterobacterales in the EU/EEA is high to very high because of continued transmission of high-risk hospital lineages, plasmid-mediated dissemination of carbapenemase genes, and outbreaks across healthcare networks (Lowe et al., 2022). In parallel, WHO notes that carbapenem-resistant *Pseudomonas aeruginosa* and methicillin-resistant *Staphylococcus aureus* remain high-priority pathogens because of their substantial burden in healthcare settings, whereas vancomycin-resistant *Enterococcus faecium* is prioritized partly because of its capacity to transmit resistance elements (Sati et al., 2025). In sub-Saharan Africa, laboratory access remains a major bottleneck: in a 14-country analysis, laboratories with AST capacity collectively provided geographical access to less than 50% of the general population in seven countries (Hayward and Turner, 2022). Together, these data support a strong rationale for rapid, decentralized, and context-adapted point-of-care (POC) diagnostics directed toward WHO priority pathogens, especially in settings where delayed laboratory confirmation amplifies mortality, transmission, and inappropriate antibiotic exposure.

Emerging POC platforms show promise in stratified contexts. Lateral flow assays detecting *mecA* or *blaKPC* genes provide visual readouts in 90% concordance to reference methods (Shanmugakani et al., 2020; Gopal et al., 2021). Similarly, CRISPR-Cas–based microfluidic biosensors can identify resistance markers in whole blood without conventional nucleic acid extraction. Because CRISPR-Cas detection relies on guide RNA-programmed recognition of target nucleic acids and signal generation through Cas-mediated reporter cleavage, it is particularly well suited to miniaturized and closed-chip diagnostic formats. This is a critical advantage in settings lacking centrifugation or cold-chain logistics (Gopal et al., 2021). Single-cell diagnostics, though still largely experimental, enable phenotypic AST at the individual bacterial level within hours, potentially obviating the need for population-level growth altogether (Li et al., 2023). However, successful deployment requires more than technological innovation—it demands context-aware design. A POC test for CRE must be affordable. Moreover, integration into national AMR surveillance networks is essential to transform isolated diagnoses into public health intelligence. **Table 1** selected WHO BPPL 2024 pathogens most relevant to rapid microfluidic AMR diagnostics, together with their public health rationale, epidemiological relevance, and priority diagnostic needs.

**Table 1:** WHO BPPL pathogens most relevant to rapid microfluidic AMR diagnostics, together with their public health rationale and diagnostic priorities.

WHO BPPL tier	Pathogen–resistance combination	Why prioritized	Regional/epidemiological relevance	Priority rapid diagnostic need	References
Critical	Carbapenem-resistant *Acinetobacter baumannii*	High mortality, very limited treatment options, strong healthcare-associated transmission potential	Major burden in ICUs and other high-risk hospital settings; representative of last-resort antibiotic resistance in Gram-negative pathogens	Rapid identification plus direct phenotypic AST for severe hospital-acquired infection	(Sati et al., 2025; Wise, 2025)
Critical	Third-generation cephalosporin-resistant Enterobacterales	Very high global burden; major cause of sepsis, urinary tract infection, and bloodstream infection; narrowing empiric treatment options	WHO reported that >40% of *Escherichia coli* and >55% of *Klebsiella pneumoniae* globally were resistant to third-generation cephalosporins in 2023, exceeding 70% in the African Region	Direct-from-specimen detection with early resistance profiling and streamlined AST	(Jesudason, 2024; Sati et al., 2025)
Critical	Carbapenem-resistant Enterobacterales	High attributable mortality and strong outbreak potential due to plasmid-mediated carbapenemase dissemination	ECDC has assessed the probability and impact of further CRE spread in the EU/EEA as high to very high; rising incidence of carbapenem-resistant *K. pneumoniae* bloodstream infection has been documented in Europe	Rapid carbapenemase-oriented testing plus confirmatory phenotypic AST	(Lowe et al., 2022; Graham et al., 2024)
Critical	Rifampicin-resistant *Mycobacterium tuberculosis*	Very high burden and major treatment implications; delayed resistance detection leads to prolonged transmission and poor outcomes	Especially relevant in high-TB-burden settings and settings requiring decentralized testing	Near-patient molecular resistance testing from sputum-compatible workflows	(Sati et al., 2025; Wise, 2025)
High	Vancomycin-resistant *Enterococcus faecium*	Important nosocomial pathogen with difficult treatment and capacity to transmit resistance determinants	Persistent burden in healthcare settings; included by WHO among pathogens requiring targeted public-health intervention	Ward-level screening and rapid susceptibility/resistance testing in hospital settings	(Sati et al., 2025; Wise, 2025)
High	Carbapenem-resistant *Pseudomonas aeruginosa*	Difficult-to-treat healthcare-associated infection with substantial ICU relevance	WHO moved CRPA from critical to high in 2024 but emphasized that its burden remains significant in some regions and healthcare settings	Rapid hospital-based AST and resistance profiling for ventilator-associated and bloodstream infection contexts	(Sati et al., 2025; Wise, 2025)
High	Methicillin-resistant *Staphylococcus aureus* (MRSA)	Persistent healthcare burden and ongoing transmission risk; clinically important in bloodstream, wound, and device-associated infections	ECDC estimated 4.48 MRSA bloodstream infections per 100, 000 population in the EU in 2024; CDC reported that MRSA was one of the major hospital-onset AMR threats monitored during and after the COVID-19 period	Rapid screening, species identification, and targeted AST to support de-escalation and infection control	(Cartwright et al., 2024; Graham et al., 2024)
High	Fluoroquinolone-resistant *Salmonella Typhi*/non-typhoidal *Salmonella*	High enteric disease burden, especially in low- and middle-income countries (LMICs); resistance complicates syndromic treatment and outbreak control	WHO specifically highlights Salmonella as a high-priority burden in LMICs; typhoid remains concentrated in endemic low- and middle-income settings, especially South Asia and parts of Africa	Low-cost multiplex fever/enteric pathogen testing with resistance-marker integration	(Kuehn et al., 2025)

Addressing these stratified needs is not merely equitable—it is epidemiologically essential. As AMR respects no borders, diagnostic gaps in one region become therapeutic crises in another through travel and trade. Thus, the development and equitable distribution of POC diagnostics for WHO priority pathogens constitute a non-negotiable pillar of global AMR containment.

## 3 Evolution of microfluidic architectures for bacterial detection and resistance profiling

### 3.1 Milestones in device miniaturization and automation

Microfluidic technology refers to the precise manipulation and control of very small volumes of fluids within micro-scale channels, chambers, or droplets. In AMR diagnostics, this technology enables miniaturized integration of sample preparation, bacterial enrichment, nucleic acid amplification, sensing, and susceptibility testing within compact platforms. Microfluidic technologies for AMR diagnosis have progressed from early proof-of-concept devices focused on bacterial trapping, separation, or growth monitoring to increasingly integrated lab-on-a-chip platforms that can operate in near-patient settings (Ferrara et al., 2024; Hou et al., 2025; Jesudason, 2026). Early microfluidic devices in the 2000s primarily focused on bacterial trapping or separation using hydrodynamic forces or dielectrophoresis, but lacked integrated detection modalities and required off-chip analysis. These initial designs, while innovative, suffered from poor reproducibility, manual fluid handling, and incompatibility with complex clinical matrices such as whole blood or sputum (Kelly et al., 2020). The pivotal shift occurred in the late 2010s with the convergence of microfabrication advances, surface chemistry optimization, and embedded sensing elements, enabling true sample-in-answer-out functionality (Aluzaite et al., 2025).

A landmark milestone was the development of polymer-based microchips incorporating on-chip lysis, nucleic acid amplification, and electrochemical detection within a monolithic architecture. For instance, a 2022 platform utilizing cyclic olefin copolymer (COC) demonstrated simultaneous detection of *mecA*, *vanA*, and *blaKPC* genes directly from positive blood cultures in under 90 minutes, with 98% concordance to standard PCR. This integration eliminated cross-contamination risks associated with tube-to-tube transfers and reduced operator dependency—a critical factor in resource-limited environments. Further miniaturization was achieved through digital microfluidics (DMF), where discrete droplets are manipulated via electrowetting-on-dielectric (EWOD) actuation. DMF platforms have enabled programmable workflows for multiplexed AST, including gradient antibiotic exposure and real-time impedance monitoring of bacterial growth inhibition at sub-microliter volumes (Akuoko et al., 2022).

**Figure 2:**
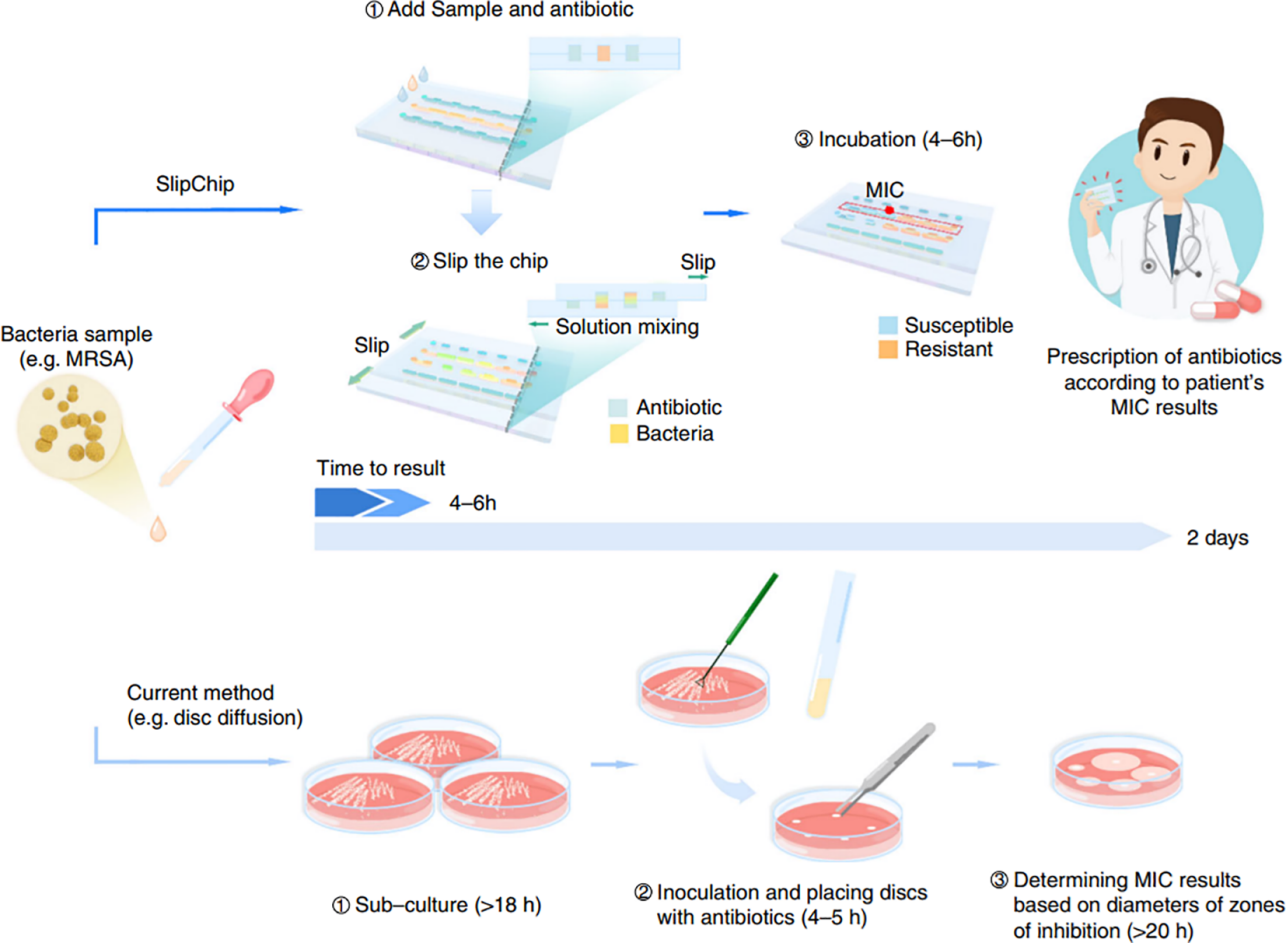
Schematic comparison of the self-dilution for faster antimicrobial susceptibility testing (SDFAST) workflow and a conventional disk diffusion workflow. The figure illustrates differences in antibiotic dilution strategy, operational steps, and readout mode. Reproduced with permission from (Wat et al., 2025).

Automation has been equally transformative. Early microfluidic AST relied on optical density measurements requiring bulky external readers, but recent iterations embed miniaturized photodiodes, complementary metal-oxide-semiconductor (CMOS) sensors, or plasmonic nanostructures that transduce bacterial metabolic activity into quantifiable electrical signals (Ripandelli et al., 2024). As shown in **Figure 1**, researchers have developed a microfluidic device called Self Dilution for Faster Antimicrobial Susceptibility Testing (SDFAST). This SlipChip-based device consists of two layers of microchips. By simply connecting the two chips, bacterial suspensions and antibiotics can be injected. With a single press of the microchip, one microchip can slide over the other, diluting the antibiotics within seconds and thoroughly mixing them with the bacterial samples. By combining SDFAST with the water-soluble tetrazolium salt 8 (WST-8) assay, a variety of clinically relevant bacteria were tested under different antibiotics, including *Acinetobacter baumannii*, *Escherichia coli*, *Klebsiella pneumoniae*, and *Staphylococcus* spp. Color analysis after 4–6 hours of incubation showed a sudden change in the color of WST-8 in certain wells following the dilution of antibiotics, demonstrating the immediate identification of the minimum inhibitory concentration (MIC) without the need for instrumentation. The assay was evaluated using 51 clinical isolates and showed 92% concordance with the reference method, supporting the analytical accuracy of the reported SDFAST platform (Wat et al., 2025). **Figure 2** provides a schematic comparison of the SDFAST workflow and a conventional disk diffusion workflow. This comparison is based on differences in operational steps, on-chip antibiotic dilution, and readout strategy rather than on a direct quantitative comparison of diagnostic performance. Such innovations not only reduce instrument footprint and operational complexity, but also improve the feasibility of decentralized and point-of-care AST.

### 3.2 Materials used in microfluidic devices and their impact on antibiotic adsorption

Material selection is a fundamental design variable in microfluidic devices because it influences not only fabrication strategy and cost, but also assay fidelity, analyte recovery, and clinical translatability. Polydimethylsiloxane (PDMS) remains widely used in academic microfluidics because of its optical transparency, gas permeability, elastomeric properties, and compatibility with soft lithography (Grindulis et al., 2025). However, PDMS is also known to adsorb and absorb hydrophobic small molecules, including drugs, which can reduce the effective concentration of antibiotics within microchannels and thereby bias phenotypic AST results (Agha et al., 2022). This limitation is particularly important in assays that rely on precise drug exposure, prolonged incubation, or quantitative determination of minimum inhibitory concentrations. In this context, material choice is directly linked to analytical accuracy rather than being a purely engineering consideration.

By contrast, thermoplastic materials such as COC, cyclic olefin polymer (COP), and polymethyl methacrylate (PMMA) are increasingly favored for translational microfluidic platforms. Compared with PDMS, these materials generally exhibit lower small-molecule sorption, greater dimensional stability, and better compatibility with scalable manufacturing approaches such as injection molding and hot embossing (Amid et al., 2022). These properties make them attractive for disposable cartridges and integrated sample-to-answer systems intended for near-patient or point-of-care deployment. Glass and silicon also remain important material platforms because they provide excellent chemical resistance, thermal stability, and low background interaction with many assay reagents (Aralekallu et al., 2023). These characteristics are advantageous for applications involving high-temperature amplification, solvent-intensive workflows, or integrated sensing components, although fabrication and bonding are typically more complex and costly than for polymer-based systems.

Importantly, no single material is ideal for all AMR applications. PDMS is still valuable for rapid prototyping, gas-permeable cell-based studies, and mechanically tunable experimental platforms. However, for antibiotic exposure studies and phenotypic AST, materials with lower drug sorption, such as COC/COP, PMMA, or glass-based hybrids, are often preferable because they better preserve the intended antibiotic concentration and improve assay reproducibility (Khan et al., 2025). Surface modifications and antifouling coatings can reduce nonspecific adsorption, but they do not fully eliminate bulk absorption in PDMS (Nge et al., 2013). Therefore, future microfluidic platforms for AMR diagnostics should treat material selection as a core determinant of analytical performance, manufacturing scalability, and regulatory robustness rather than as a secondary fabrication detail.

### 3.3 Engineering closed systems for minimal operator intervention and contamination risk

**Figure 3:**
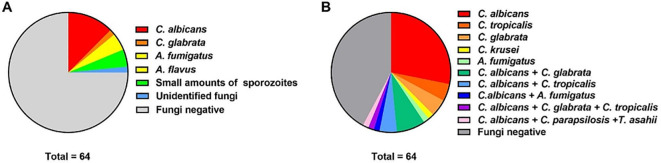
Detection of pathogenic fungi in patients with respiratory tract infections using culture methods and microfluidic chips. **(A)** Results of microbial culture. **(B)** Analysis by microfluidic chip. Reproduced with permission from (Yao et al., 2024).

The clinical utility of any diagnostic platform hinges on its ability to function reliably outside controlled laboratory environments, particularly in acute care or POC settings where trained personnel and sterile conditions are scarce. This necessity has driven the engineering of fully closed, sample-to-answer microfluidic systems that encapsulate the entire diagnostic cascade—from specimen introduction to result interpretation—within a single, disposable cartridge. Such architectures minimize human intervention, thereby reducing both procedural errors and biohazard exposure, while simultaneously preventing amplicon carryover contamination that plagues open-tube molecular assays. Modern closed-system designs employ multi-layered fluidic networks with passive valving mechanisms (e.g., capillary burst valves, hydrophobic barriers) that sequentially release reagents in response to centrifugal force or pneumatic pressure (Gamage et al., 2025). Contamination control is further enhanced through material science innovations. Cartridges fabricated from cyclic olefin polymer (COP) exhibit ultra-low DNA adsorption, preserving nucleic acid yield, while internal surfaces are functionalized with UV-curable antifouling coatings to prevent biofilm formation during storage (Baltas et al., 2024). Some systems incorporate built-in negative controls—such as lyophilized water compartments that undergo identical processing—to flag reagent degradation or manufacturing defects. Additionally, waste chambers are hermetically sealed post-assay, complying with biosafety level 2 (BSL-2) disposal protocols without requiring secondary containment. As shown in **Figure 3**, Yao et al. used a fully enclosed microfluidic chip technology to rapidly detect the DNA of 20 fungal spectra in 64 clinical samples from coastal patients, which was then verified by Real-time qPCR and analyzed in conjunction with their clinical baseline data. The microfluidic chip results showed that 36 cases were infected with *Candida* spp., and 27 cases tested negative for fungi, consistent with the verification by real-time qPCR. In contrast, only 16 fungal infections were detected by culture methods; however, one culture-positive sample tested negative by microfluidic chip and qPCR verification. These findings suggest that microfluidic chip technology based on isothermal amplification combined with routine blood tests can improve the speed and accuracy of identifying respiratory fungal pathogens (Yao et al., 2024). Crucially, these gains were sustained across diverse operator skill levels, underscoring the robustness of automation. Moreover, the elimination of manual pipetting steps decreased hands-on time from 45 minutes to less than 2 minutes per test, freeing staff for other critical duties.

From a public health perspective, closed cartridges facilitate decentralized surveillance. Each device can embed RFID or QR codes that log test type, lot number, and anonymized resistance profiles, enabling real-time aggregation into national AMR databases. This capability transforms individual diagnostics into epidemiological sentinels, allowing health authorities to detect emerging resistance clusters before they escalate into outbreaks.

### 3.4 Single-cell resolution platforms

Conventional bulk diagnostics mask critical biological heterogeneity by reporting population-averaged resistance phenotypes, thereby overlooking rare but clinically significant resistant subpopulations that drive treatment failure and resistance propagation. Single-cell microfluidic platforms have emerged to address this limitation, offering unprecedented resolution in profiling antimicrobial susceptibility at the individual bacterial level (Barakat et al., 2026). These technologies exploit microfabricated traps, droplet microfluidics, or nanowell arrays to isolate single cells, followed by real-time monitoring of growth dynamics or metabolic responses under antibiotic pressure. One pioneering approach utilizes mother machine architectures—microchannels that trap bacteria while permitting continuous media perfusion—to track lineage-specific responses over multiple generations (Cheng et al., 2025). In a reported study, this method revealed that *Pseudomonas aeruginosa* populations exposed to ciprofloxacin contained persister subclones exhibiting transient tolerance despite lacking known resistance genes; these persisters regenerated full populations upon antibiotic withdrawal, explaining recurrent infections (Ábrahám et al., 2024). Similarly, droplet-based platforms encapsulate single bacteria with fluorescent viability dyes and antibiotics in picoliter-volume emulsions. High-throughput imaging then quantifies division events or membrane integrity loss, generating susceptibility distributions rather than binary resistant/susceptible calls (Zhang et al., 2025). Phenotypic heterogeneity is particularly consequential in chronic infections like cystic fibrosis or prosthetic joint infections, where biofilms harbor metabolically dormant cells impervious to conventional AST. Single-cell impedance cytometry integrated into microfluidic chips can detect subtle changes in membrane capacitance upon antibiotic exposure, identifying tolerant phenotypes missed by optical methods (Ábrahám et al., 2024). Researchers have developed the LM-ResiChip, a portable microfluidic chip-based qPCR assay for detecting stress-resistant Listeria monocytogenes. Rather than broadly targeting unspecified stress-related genes, this platform specifically targets four pangenome-informed molecular markers—*dasC, malL, gtfA*, and *thiE*—that were identified in stress-resistant strains and are associated with adaptation to salt, oxidative, and nutrient stress conditions. The assay was implemented on a compact qPCR system with a 3D polymer-based chip, enabling simultaneous detection of all four targets in a single run. The LM-ResiChip demonstrated high specificity, amplifying only stress-resistant L. monocytogenes isolates without cross-reactivity with stress-sensitive strains or other Listeria spp (Kim et al., 2025). The clinical value of single-cell resolution lies in its capacity to inform precision dosing. By quantifying the proportion of resistant variants and their MIC, clinicians can tailor combination regimens or adjust dosing intervals to suppress minority clones. The following **Table 2** compares performance characteristics of bulk versus single-cell AST platforms (Ábrahám et al., 2024; Cheng et al., 2025; Barakat et al., 2026).

**Table 2:** Comparison of performance characteristics between batch and single-cell AST platforms.

Feature	Bulk culture/PCR	Single-Cell microfluidics
Detection Limit for Resistant Subclones	1%	0.01%
Turnaround Time	24–72 h	4–8 h
Phenotypic vs. Genotypic Data	Either/or	Simultaneous
Biofilm-Compatible	No	Yes
Cost per Test	$10–20	$50–80

While cost and complexity remain barriers to routine adoption, ongoing efforts to simplify readout (e.g., machine learning–assisted image analysis) and scale manufacturing are narrowing the gap. As evidence mounts that minority resistant populations dictate long-term outcomes, single-cell microfluidics transition from research curiosities to essential tools in the antimicrobial stewardship arsenal.

## 4 Bridging genotypic and phenotypic diagnostics through microfluidic innovation

### 4.1 Real-time phenotypic assays

**Figure 4:**
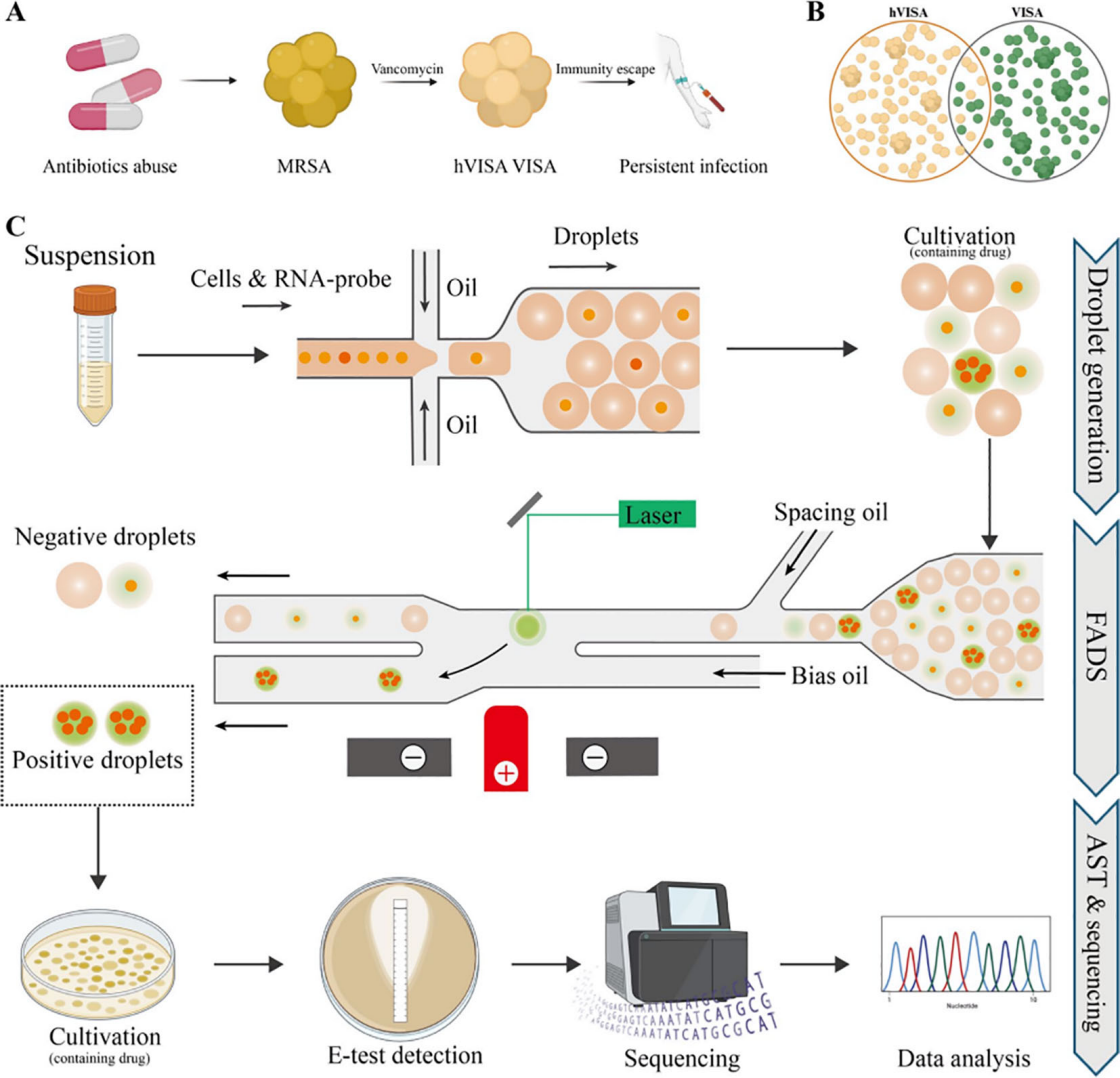
Emergence, definition, and screening process of heterogeneous vancomycin-intermediate *Staphylococcus aureus* (hVISA). **(A)** hVISA is thought to arise under antibiotic selection pressure, particularly vancomycin, during methicillin-resistant *Staphylococcus aureus* (MRSA) infections. **(B)** hVISA consists predominantly of vancomycin-susceptible bacterial cells but contains a minor subpopulation with intermediate resistance to vancomycin. **(C)** Workflow for ultra-high-throughput screening of hVISA based on fluorescence-activated droplet sorting (FADS). Reproduced with permission from (Cheng et al., 2025).

The clinical imperative to move beyond static susceptibility reports toward dynamic, functional assessments of bacterial behavior under antibiotic stress has catalyzed the development of real-time phenotypic microfluidic assays. Unlike conventional broth microdilution, which yields a single minimum inhibitory concentration (MIC) value after 16–24 hours, microfluidic platforms enable continuous, high-resolution monitoring of individual bacterial cells or microcolonies as they respond to antimicrobial agents (Juliet et al., 2024). This temporal granularity reveals critical kinetic parameters—such as lag time extension, division rate suppression, and morphological adaptation—that are invisible to endpoint assays but highly predictive of therapeutic outcome. Central to this capability is the integration of multimodal sensing within microchannels (Chen et al., 2023). **Figure 4** illustrated the emergence, definition, and screening process of heterogeneous vancomycin-intermediate *Staphylococcus aureus* (hVISA). A microfluidic platform based on fluorescence-activated droplet sorting (FADS) has been introduced for the rapid detection and isolation of heterogeneous vancomycin-intermediate *Staphylococcus aureus* (hVISA). Based on the Poisson distribution theoretical model, the bacterial suspension concentration was optimized to 10–^6^ CFU/mL, achieving a single-bacterium droplet encapsulation rate of about 30%. The selected fluorescent probe showed detectable fluorescence signals as early as 6 hours after cultivation, with stability maintained for at least 1 week. Drug concentration validation indicated that 4 μg/mL vancomycin could effectively inhibit susceptible bacteria without interfering with fluorescence detection. The platform successfully isolated the resistant subpopulation from the hVISA reference strain Mu3 within 12 hours and further validated its efficacy using 15 clinical hVISA isolates. This study innovatively integrated a microfluidic detection platform with FADS to achieve rapid detection of hVISA (Cheng et al., 2025).

Electrochemical transduction offers label-free alternatives. Impedance cytometry measures changes in bacterial membrane capacitance or cytoplasmic conductivity upon antibiotic exposure. Researchers have optimized the metagenomic nanopore sequencing and bioinformatics pipeline for the detection of pathogens and AMR genes in urine samples. Among the 10 nanopore sequencing false-positive samples, six samples may have reflected latent infections because they were from patients with UTI risk factors and clinical symptoms. Moreover, nanopore sequencing had a much higher detection rate for mixed urinary tract infections (two or more microorganisms) than urine culture (33.51% vs. 5.81%, *p* <.01) (Manjiao et al., 2023). These electrical readouts are inherently compatible with miniaturized electronics, enabling portable POC devices. Such insights are vital for guiding combination therapy or extended infusions. Moreover, real-time data feed pharmacokinetic/pharmacodynamic (PK/PD) models, allowing clinicians to simulate dosing regimens that suppress resistant mutants—a cornerstone of precision antimicrobial stewardship.

### 4.2 On-chip nucleic acid amplification

While phenotypic assays reveal functional resistance, genotypic methods offer unparalleled speed and specificity for known resistance mechanisms. Microfluidic innovation has thus focused on integrating nucleic acid amplification directly onto chips, transforming molecular detection from a multi-step laboratory procedure into a streamlined, contamination-resistant process. Two primary strategies dominate: miniaturized thermal cycling for PCR and isothermal amplification leveraging enzymatic reactions at constant temperature—each with distinct trade-offs in speed, complexity, and multiplexing capacity (Chengzhuang et al., 2022).

PCR-based microfluidics benefit from decades of optimization in primer design and thermocycling protocols. Recent chips embed thin-film heaters and temperature sensors into polymer (e.g., COP, PMMA) or silicon substrates, achieving ramp rates >10 °C/s and cycle times under 30 minutes. Crucially, closed-system design prevented amplicon carryover, a persistent issue in open-tube workflows. Digital PCR (dPCR) variants further enhance sensitivity: partitioning samples into thousands of nanoliter reactors enables absolute quantification of low-abundance targets (e.g., emerging carbapenemases in surveillance samples) without standard curves (Bin et al., 2022).

**Figure 5:**
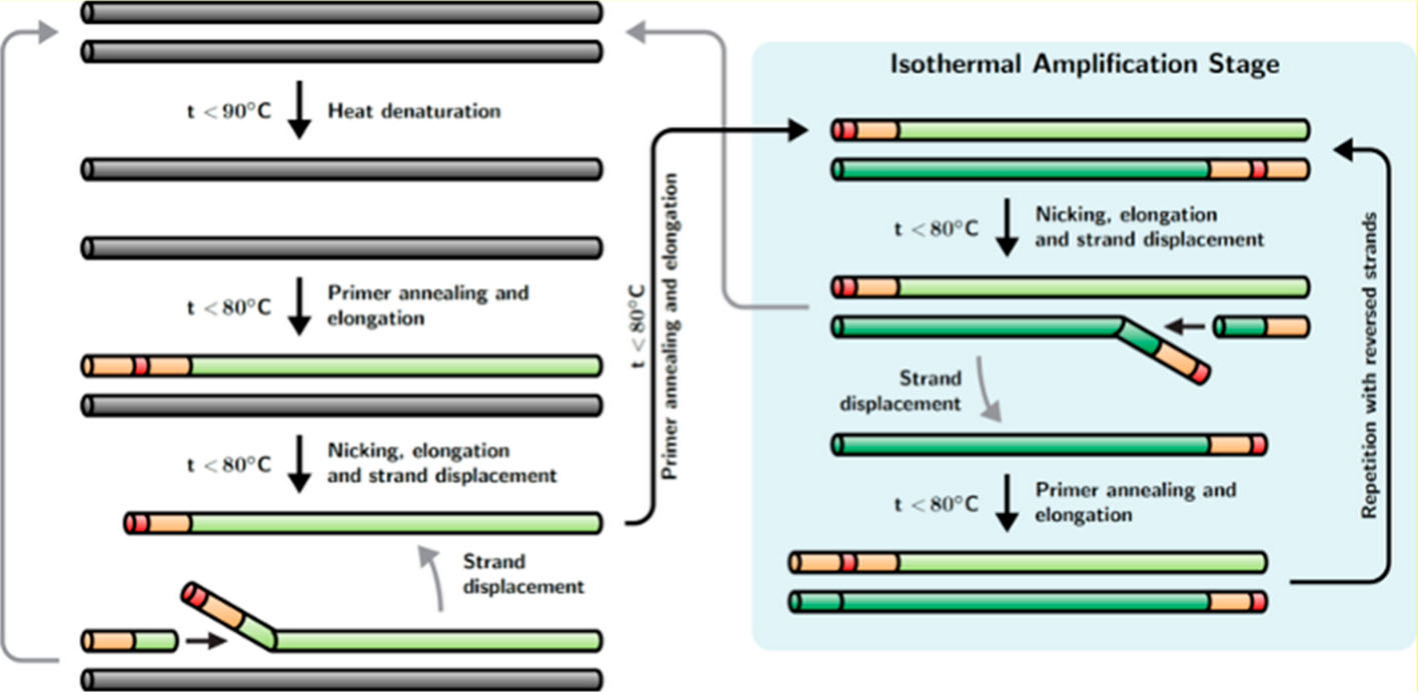
Schematic diagram of strand displacement chain reaction (SDCR) amplification. Reproduced with permission from (Smirnova et al., 2025).

Isothermal amplification methods—particularly loop-mediated isothermal amplification (LAMP) and recombinase polymerase amplification (RPA)—are well suited to resource-limited settings because they operate at constant temperature and therefore require simpler instrumentation than conventional PCR (Bin et al., 2022). Microfluidic LAMP platforms often use wax valves or capillary forces to sequentially release lyophilized reagents upon sample introduction. Beyond these established isothermal approaches, newer hybrid amplification strategies have also emerged to combine the rapid kinetics of isothermal amplification with selected features of PCR. One representative example is strand displacement chain reaction (SDCR), which has been described as a hybrid amplification technique based on PCR and isothermal nucleic acid amplification (Smirnova et al., 2025). In a comparative study using human, bacterial, and viral genetic materials, SDCR showed high sensitivity and amplification efficiency and achieved a greater amplification factor than conventional PCR while reducing the required number of cycles (Smirnova et al., 2025). **Figure 5** illustrates the principle of SDCR amplification. By positioning SDCR within this broader amplification framework, it becomes clear that SDCR is discussed here not as an isolated method, but as an emerging hybrid strategy relevant to rapid nucleic acid detection and future microfluidic diagnostic development. However, isothermal assays face challenges in multiplexing due to primer interference and non-specific amplification, limiting their scope compared to PCR. Hybrid approaches are emerging to leverage the strengths of both paradigms. CRISPR-Cas systems integrated post-amplification provide sequence-specific detection: Cas12a cleavage of fluorescent reporters upon target recognition adds a layer of specificity that reduces false positives from non-specific amplification (Rajeshwari and Karuna, 2022).

For on-chip nucleic acid testing, material compatibility remains critical because surfaces must minimize nucleic acid loss during lysis, extraction, and amplification. COP-based and related thermoplastic substrates generally exhibit low DNA binding, and internal coatings such as bovine serum albumin (BSA) or Pluronic F-127 can further reduce analyte loss (Jahromi et al., 2020). Validation studies confirm that on-chip extraction yields >90% of input DNA from complex samples like stool or wound swabs, rivaling manual kits (Juliet et al., 2024). As regulatory frameworks evolve (e.g., FDA clearance for microfluidic AMR panels), these integrated systems are poised to replace sequential culture and PCR workflows in hospital microbiology labs.

## 5 Performance validation against clinical benchmarks

### 5.1 Sensitivity and specificity in complex matrices

The diagnostic fidelity of microfluidic AMR platforms hinges critically on their ability to maintain high analytical sensitivity and specificity when deployed in clinically relevant, complex biological matrices such as whole blood, urine, and sputum. These specimens present formidable challenges—including high viscosity, cellular debris, protein fouling, and variable pH—that can interfere with both nucleic acid extraction and phenotypic readouts. Rigorous validation against gold-standard clinical benchmarks is therefore essential to establish clinical utility.

Urine, though less complex than blood, introduces variability through osmolarity, pH, and crystalline precipitates that can clog microchannels or quench fluorescence signals. Hydrophilic polymer coatings (e.g., PEG-diacrylate) prevent nonspecific adsorption, while inline filtration membranes (pore size 0.45 µm) remove particulates without losing bacterial cells. In a head-to-head comparison using 200 clinical urine isolates, a droplet digital PCR (ddPCR)-integrated chip detected *vanA* in enterococci with 99.1% concordance to broth microdilution, even in samples with >50 white blood cells/HPF—a condition that typically causes false negatives in lateral flow assays (Yuanhui et al., 2020).

Sputum poses the greatest challenge due to its viscoelastic mucus network, which entraps bacteria and impedes diffusion. Successful microfluidic strategies incorporate on-chip mucolysis—typically using dithiothreitol (DTT) or N-acetylcysteine (NAC)—followed by size-exclusion sorting (Cheng et al., 2024). A spiral inertial microchannel device processed liquefied sputum at 2 mL/min, enriching *Mycobacterium tuberculosis* by 100-fold while removing >95% of human DNA. When coupled with loop-mediated isothermal amplification (LAMP), this system detected rifampicin resistance (*rpoB* mutations) with 92.5% sensitivity and 97.3% specificity versus Xpert MTB/RIF, with a limit of detection of 100 bacilli/mL (Krishnan et al., 2021). A finding with direct implications for combination therapy selection. The following **Table 3** summarizes performance metrics of representative microfluidic platforms across key clinical matrices.

**Table 3:** Performance indicators of microfluidic platforms in the clinical field.

Matrix	Platform type	Sensitivity (%)	Specificity (%)	Limit of detection	References
Urine	Centrifugal PCR	96.2	98.7	500 CFU/mL	(Yuanhui et al., 2020)
Sputum	ddPCR-on-chip	92.5	97.3	10² CFU/mL	(Krishnan et al., 2021)

These data underscore that microfluidic systems, when engineered with matrix-specific preprocessing, can match or exceed conventional laboratory methods in diagnostic accuracy. Importantly, they do so while preserving the viability of pathogens for downstream phenotypic characterization—a dual capability that enhances clinical decision-making in sepsis and other acute infections.

### 5.2 Turnaround time reduction

Accelerating the diagnostic timeline from specimen collection to actionable result represents one of the most compelling clinical advantages of microfluidic AMR platforms. Traditional workflows—encompassing culture, colony isolation, identification, and susceptibility testing—typically require 48–72 hours, during which empiric broad-spectrum antibiotics are administered, often inappropriately. Microfluidic integration compresses this cascade into a single, automated process that delivers genotype-phenotype correlations in under 4 hours, with some modalities achieving results in under 60 minute (Yuanhui et al., 2020).

To address this, an innovative microfluidic biosensor that uniquely combines chemotaxis-based guidance with impedance cytometry for the selective and sensitive detection of single zoospores has been developed. Experimental validation demonstrated robust single-zoospore detection capability. The system achieved a signal-to-noise ratio (SNR) of approximately 5.9 when zoospores were purely chemotactically drawn into the channel. The SNR could be enhanced to about 17 when a minimal carrying flow was introduced to assist the process. This work provides a compelling proof-of-concept for a portable, low-cost, and remotely operable sensing platform, highlighting its significant potential for real-time, in-field pathogen monitoring and early-warning systems in agriculture and ecosystem conservation (Zhang et al., 2025).

In a similar vein, the AM-DMF-ddRPA platform combines active-matrix digital microfluidics with digital RPA to achieve fully automated, absolute nucleic acid quantification. The process from sample to result is completed in under 60 minutes, representing a significant leap in automation and speed for digital nucleic acid analysis (Ji et al., 2025). For phenotypic susceptibility testing, a self-priming digital microfluidic chip has been developed for single-cell AST. By utilizing integrated microvalves for self-digitization, this ready-to-use chip eliminates the need for bulky external pumps. It performs AST at the single-cell level and delivers results within 6 hours, offering a practical tool for POC applications (Pang et al., 2025). Moving closer to a complete diagnostic solution, a Static Droplet Array (SDA) chip-based system tackles the entire diagnostic cascade for bloodstream infections. This method can perform quantitative detection, identification, and AST directly from untreated whole blood in approximately 12 hours. It employs AI-powered image analysis to determine MIC, with results showing over 95% agreement with standard clinical methods (Diao et al., 2025). The **Table 4** below summarizes four representative recent studies, illustrating how they achieve rapid detection.

**Table 4:** Performance indicators of microfluidic platforms in the clinical field.

Platform/Chip name	Core technology & integration	Turnaround time	Key performance & Clinical validation	References
New type of microfluidic impedance detection sensor	Microfluidic impedance cytometer combined with chemotactic guidance	Real-time/Near real-time monitoring	Single-zoospore detection with a signal-to-noise ratio (SNR) of ~5.9 (pure chemotaxis) and ~17 (with assisted flow)	(Zhang et al., 2025)
Self-Priming DMF Chip	DMF with self-priming microvalves for single-cell AST; no external pumps needed.	6 hours	Enables phenotypic AST at the single-cell level. Designed as a ready-to-use platform for point-of-care testing.	(Ji et al., 2025)
AM-DMF-ddRPA	Active-Matrix DMF integrated with digital RPA for absolute quantification.	< 60 minutes	Achieves full automation from nucleic acid extraction to result analysis. Showcased for rapid viral detection.	(Pang et al., 2025)
Static Droplet Array (SDA) Chip	Microcavity array for single-bacteria isolation, combined with MALDI-TOF ID and AI-based AST.	~12 hours	Processes untreated whole blood. Performs quantification, ID, and AST. MIC results >95% agreement with standard methods.	(Diao et al., 2025)

## 6 Synergistic integration of emerging technologies to enhance detection fidelity

### 6.1 CRISPR-Cas systems coupled with microfluidics

CRISPR-Cas–based diagnostics rely on guide RNA-programmed Cas nucleases to recognize predefined nucleic acid targets with high sequence specificity. In diagnostic formats, Cas12 and Cas13 are especially important because, after target recognition, they can activate collateral cleavage of nearby single-stranded reporter molecules (Menofy et al., 2025). This property allows target binding to be converted into measurable fluorescent, colorimetric, lateral-flow, or electrochemical signals. In the context of AMR, guide RNAs can be designed against resistance determinants such as *mecA, vanA, blaNDM*, or *blaKPC*, or against species-specific targets linked to resistance profiling (Bing et al., 2022). Thus, the analytical value of CRISPR-Cas in AMR diagnostics lies in its ability to provide rapid genotypic recognition with high sequence discrimination.

Microfluidics strengthens this strategy not by replacing CRISPR chemistry, but by organizing the upstream and downstream workflow into a miniaturized, closed, and automated format. A microfluidic cartridge can meter samples and reagents, perform lysis or nucleic-acid preparation, integrate pre-amplification when needed, partition reactions into isolated chambers, and transfer amplicons to a detection zone while minimizing carryover contamination (Trinh and Lee, 2021). The use of nanoliter- to microliter-scale compartments also reduces reagent consumption, shortens diffusion distances, and improves the practicality of point-of-care testing. A representative example is the Lift-CM platform, which integrates isothermal amplification and CRISPR/Cas12a detection within a centrifugal microfluidic cartridge, physically separates amplification and detection regions to reduce amplicon contamination, and completes the workflow in approximately 30 minutes under smartphone-assisted control (Zhu et al., 2025).

For AMR applications, the key advantage of CRISPR-microfluidic coupling is the ability to connect resistance-gene recognition with compact and potentially near-patient workflows (Ahmadi et al., 2020). However, important limitations remain: many CRISPR assays still depend on upstream amplification to achieve clinical sensitivity, multiplexing remains technically challenging because of guide and reporter cross-interference, and robust validation in real clinical AMR specimens is still limited compared with conventional reference methods (Natsuhara et al., 2021). For this reason, CRISPR-microfluidic systems should currently be viewed as highly promising rapid molecular platforms whose translation will depend on further simplification of sample preparation, standardization of assay design, and stronger clinical validation (Shu et al., 2025).

The clinical utility extends beyond genotyping to functional validation. By integrating CRISPR interference (CRISPRi) circuits into microchambers, researchers can transiently silence resistance genes and observe phenotypic reversion in real time. In a proof-of-concept study, *K. pneumoniae* harboring *blaKPC* was exposed to sub-inhibitory meropenem in Cas9-silenced microdroplets; loss of fluorescence from a β-lactamase reporter confirmed on-target knockdown and restored antibiotic susceptibility within 2 hours (Ahmadi et al., 2020). Such “genotype-to-phenotype” correlation bridges a critical gap in AMR management, particularly for heteroresistant populations where only a fraction of cells carry resistance plasmids. These data underscore that CRISPR-microfluidic hybrids fulfill the WHO’s ASSURED criteria for next-generation AMR diagnostics.

### 6.2 Convergent integration of CRISPR-Cas, nanopore sequencing, and microfluidics for rapid AMR diagnosis

A conceptually important next step in AMR diagnostics is the convergence of CRISPR-Cas recognition, nanopore sequencing, and microfluidic automation within a unified workflow. These technologies contribute different but complementary functions. CRISPR-based systems provide programmable sequence recognition through guide RNA-directed target binding. In diagnostic formats, Cas12- and Cas13-based systems convert target recognition into measurable signals through collateral cleavage of reporter molecules, whereas Cas9 can be used for targeted enrichment of predefined genomic regions before sequencing (Chen et al., 2018). Nanopore sequencing, in contrast, does not rely on reporter cleavage; instead, it identifies nucleic acid sequences directly by measuring characteristic electrical current changes as DNA or RNA molecules pass through a nanopore. Its major value for AMR profiling lies in real-time long-read analysis, which can reveal not only the presence of resistance genes but also their genomic context, including linkage to plasmids, integrons, and other mobile genetic elements (Gootenberg et al., 2017). Microfluidics contributes the engineering framework needed to miniaturize and automate sample preparation, reaction partitioning, target enrichment, reagent transfer, and contamination-controlled workflow integration.

From a workflow perspective, the three technologies can be combined in a staged manner. A clinical sample can first undergo microfluidic lysis, enrichment, or nucleic acid preparation. CRISPR-Cas chemistry can then be used in one of two ways: either as a rapid front-end detection module based on Cas12/Cas13 reporter cleavage, or as a targeted enrichment strategy, such as Cas9-guided cleavage and adapter ligation, to selectively recover AMR loci for nanopore sequencing (Fuhrmeister et al., 2025). Nanopore sequencing can subsequently provide real-time readout of pathogen identity, resistance determinants, and the surrounding genomic architecture (Peng et al., 2025). In this architecture, CRISPR supplies molecular selectivity, nanopore sequencing supplies rich sequence and structural information, and microfluidics supplies workflow compression and automation.

This combination is potentially superior to using any single technology alone. Compared with stand-alone CRISPR diagnostics, nanopore sequencing can extend the output from binary target detection to broader resistance profiling and genomic-context analysis. Compared with stand-alone nanopore metagenomics, CRISPR-based enrichment can improve sensitivity for low-abundance resistance genes that may otherwise be missed in untargeted sequencing. Compared with manually assembled multi-step molecular workflows, microfluidics can reduce reagent consumption, shorten hands-on time, improve biosafety, and lower amplicon carryover risk by physically separating preparation, amplification, and detection zones (Shu et al., 2025). For AMR applications, this is particularly attractive because clinically meaningful interpretation often requires both rapid recognition of high-value resistance targets and contextual information about whether those determinants are chromosomal, plasmid-borne, or linked to other mobile elements.

Successful use has already been demonstrated for several pairwise combinations that strongly support the feasibility of this broader convergence. Context-Seq showed that CRISPR-Cas9 targeted nanopore sequencing can enrich clinically important ARGs such as *blaCTX-M* and *blaTEM* while preserving long-read genomic context in complex fecal samples (Hadlock et al., 2026). Nanopore sequencing with adaptive sampling has been applied directly to positive blood cultures for species identification and AMR prediction, achieving categorical agreement above 90% for monomicrobial infections in one large clinical study (Cheng et al., 2022). Other nanopore workflows have delivered pathogen detection and AMR prediction from urine or bronchoalveolar lavage fluid within clinically relevant time windows. On the microfluidic side, integrated CRISPR/Cas12a centrifugal platforms such as Lift-CM have shown how amplification and CRISPR detection can be physically segregated within a closed cartridge to shorten turnaround time and reduce contamination (Liu et al., 2024). In parallel, microreactor-based microfluidic systems for nanopore library preparation have demonstrated that portable sample-to-sequencer automation is technically feasible, although this has so far been shown more clearly in viral sequencing than in mature AMR-focused tripartite platforms (Zhang et al., 2025).

At present, however, fully integrated CRISPR-Cas–nanopore–microfluidic platforms for routine AMR pathogen diagnosis remain limited. The current evidence base is stronger for pairwise integration than for truly unified cartridge-to-sequencer systems. Several obstacles still need to be addressed, including the difficulty of combining high-sensitivity front-end detection with sequencing-grade library preparation in the same compact device, the risk of workflow complexity offsetting portability gains, the still imperfect error profile and bioinformatics burden of nanopore sequencing, the need for robust multiplexing without guide interference in CRISPR assays, and the limited number of large prospective clinical validation studies focused specifically on AMR decision support. Thus, the most defensible current conclusion is that CRISPR-Cas, nanopore sequencing, and microfluidics form a highly promising convergent strategy for rapid AMR diagnosis, but one that is still transitioning from modular proof-of-concept toward clinically deployable integrated systems.

### 6.3 Nanomaterial-enhanced signal transduction

Nanomaterials have emerged as indispensable transducers in microfluidic AMR biosensors, dramatically amplifying detection signals through unique optical, electronic, and catalytic properties that overcome the limitations of conventional enzyme-linked assays (Natsuhara et al., 2021). Gold nanoparticles (AuNPs), quantum dots (QDs), and graphene derivatives each offer distinct advantages: AuNPs enable colorimetric readouts via localized surface plasmon resonance (LSPR); QDs provide size-tunable fluorescence with high quantum yields; and graphene-based electrodes facilitate label-free electrochemical detection with femtomolar sensitivity.

Recent research demonstrates significant progress across various nanomaterial systems. In the realm of nucleic acid detection, metal-organic frameworks (MOFs) have been engineered as sophisticated signal amplifiers. One study developed a microfluidic chip utilizing DNA-functionalized UiO-66 nanoparticles combined with the CRISPR-Cas12a system. This platform enables the capture of single cells and subsequent ultrasensitive detection of telomerase activity, a key cancer biomarker, showcasing a PCR-free, highly specific diagnostic approach with a dual amplification mechanis (Jiang et al., 2024). Similarly, AuNP-DNA conjugates designed to hybridize with *tet(M)* mRNA produced a 100-fold signal enhancement over free fluorophores due to plasmonic coupling, enabling direct detection in unprocessed urine.

Quantum dots, particularly CdSe/ZnS core-shell structures, excel in multiplexed detection owing to their narrow emission spectra and resistance to photobleaching. A microfluidic disk encoded with three QD colors simultaneously detected *blaCTX-M*, *qnrS*, and *aac(6’)-Ib-cr* in Enterobacteriaceae by spatially separating capture probes in microwells. After 30-minute incubation, a smartphone-based imager resolved emission peaks at 525, 565, and 605 nm with minimal crosstalk, yielding 96% sensitivity across 200 clinical isolates (Kwak et al., 2021). For low-abundance targets like *mcr-1* in stool, QD-streptavidin conjugates coupled with biotinylated CRISPR reporters lowered the LoD to 1 copy/µL—comparable to digital PCR but without amplification bias (Zurek et al., 2021).

**Figure 6:**
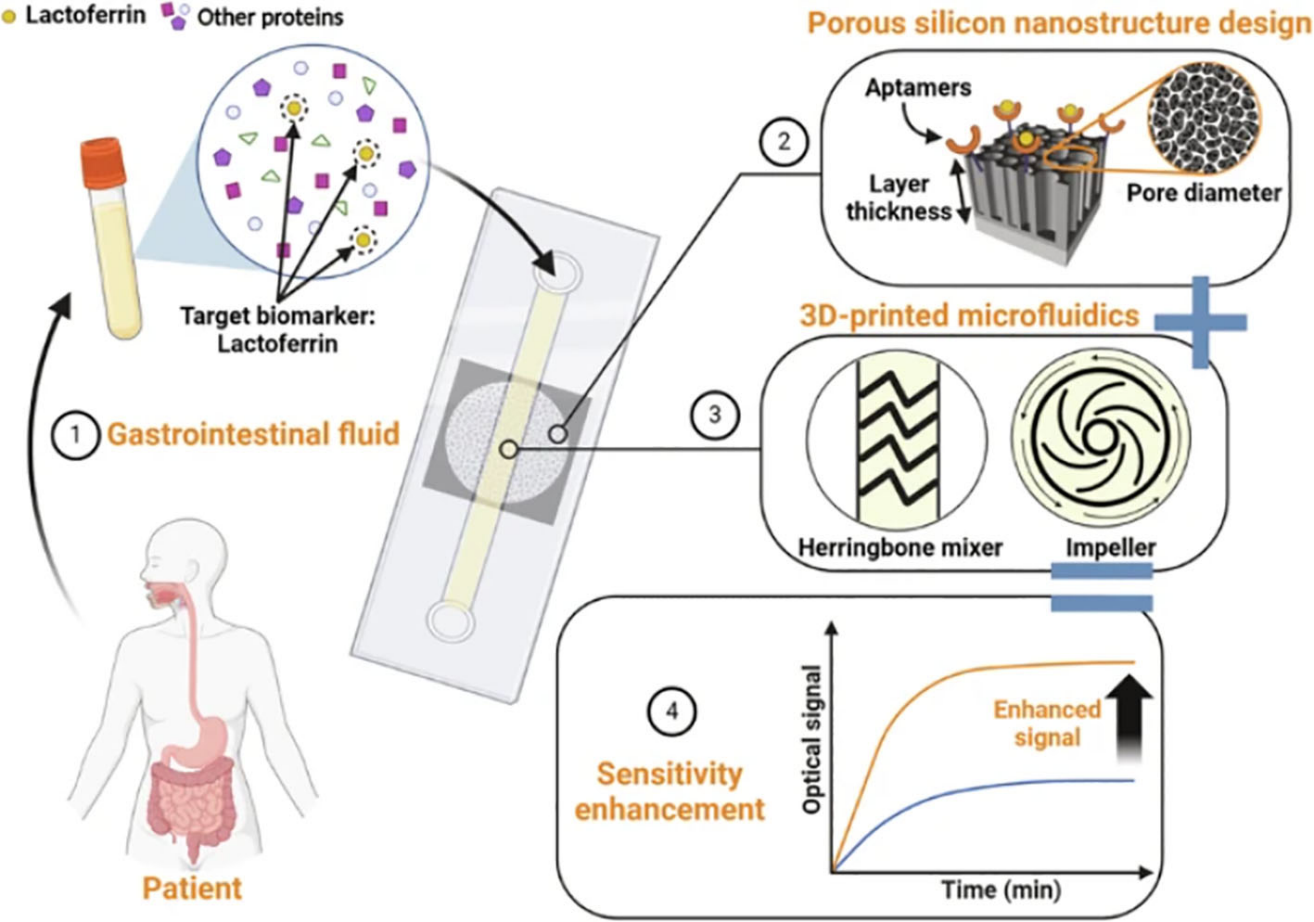
Flowchart of the development and design process of the aptamer sensor for the porous silicon (PSi) Fabry-Pérot film. Reproduced with permission from (Awawdeh et al., 2024).

For protein biomarker detection, noble metal nanocomposites are widely used to enhance electrochemical signals. A novel aptasensor for the cytokine interferon-gamma (IFN-γ) employs a nanohybrid interface of thiolated reduced graphene oxide/gold nanoparticles (TrGO-AuNPs). This design leverages a binding-induced conformational switching mechanism in a hairpin aptamer, resulting in an ultra-low detection limit (67 fg mL^-1^), a wide linear range, and a total analysis time under 45 minutes, demonstrating excellent performance in spiked human serum (Hosseini et al., 2025). Beyond metals, nanostructured porous silicon (PSi) serves as an excellent optical transducer. Research has focused on optimizing PSi Fabry-Pérot thin films by tailoring pore nanostructure and integrating them into 3D-printed microfluidic systems equipped with passive or active micromixers. This combined strategy of material and fluidic optimization significantly enhanced convection and mass transfer, achieving a limit of detection for the inflammatory biomarker lactoferrin that was over two orders of magnitude lower than previous PSi biosensors. **Figure 6** is a flowchart of the development and design process of the aptamer sensor for the porous silicon (PSi) Fabry-Pérot film (Awawdeh et al., 2024).These innovations collectively push detection limits toward single-molecule resolution while maintaining compatibility with complex clinical matrices, positioning nanomaterial-enhanced microfluidics as a cornerstone of precision antimicrobial stewardship.

### 6.4 Machine learning–driven data interpretation

The exponential growth in multidimensional data generated by integrated microfluidic platforms—spanning genomic, phenotypic, metabolic, and morphological parameters—necessitates advanced computational frameworks to extract clinically actionable insights. Machine learning (ML) algorithms, particularly deep neural networks and ensemble methods, have proven adept at deciphering complex resistance signatures that elude traditional binary classification (Mubashir et al., 2023). By training on large datasets of labeled microbial responses, these models identify subtle, nonlinear patterns predictive of resistance mechanisms, even in the absence of known genetic markers.

Convolutional neural networks (CNNs) analyze time-lapse microscopy images from microfluidic growth chambers to classify resistance based on single-cell morphology and division dynamics. A study trained a CNN on >10^6^ images of *E. coli* exposed to ciprofloxacin, using features like filamentation length, nucleoid condensation, and lysis timing to predict *gyrA* mutations with 94% accuracy—outperforming broth microdilution in speed and matching whole-genome sequencing in precision (Subedi et al., 2021). For genotypic data, random forest classifiers integrate CRISPR-Cas signal intensities across multiple resistance loci to resolve co-carriage scenarios. Li et al. developed a functional genomics approach that includes effective fusion reconstruction and sensitive cell viability and drug response assays. Using this method, they characterized approximately 100 fusion genes detected in patient samples from The Cancer Genome Atlas, identifying many activating fusions that can significantly affect sensitivity to relevant drugs and revealing the role of low-frequency fusions in promoting tumor growth (Jun et al., 2022). These advances transform microfluidic platforms from mere detection tools into intelligent diagnostic ecosystems that anticipate resistance evolution and personalize therapeutic strategies.

## 7 Translational challenges in clinical deployment and global implementation

### 7.1 Manufacturing economics

The successful translation of microfluidic diagnostics from benchtop prototypes to globally deployable medical devices hinges critically on manufacturing economics that reconcile material costs, production scalability, and clinical utility across disparate healthcare ecosystems. However, such platforms often sacrifice multiplexing capacity and quantitative accuracy, limiting their utility in complex infections like hospital-acquired pneumonia where co-resistance patterns dictate therapeutic choices. The translation of microfluidic technologies from proof-of-concept prototypes to deployable diagnostic tools is critically dependent on their manufacturing economics. A key economic driver is the adoption of robust microfabrication techniques and affordable polymer substrates. For instance, centrifugal microfluidic systems, which integrate complex fluidic operations like rapid bacterial enrichment and culture on a single chip, can be mass-produced using polymers like cyclic olefin copolymer (COC) through injection molding (Xu et al., 2024). Similarly, self-priming digital microfluidic (DMF) chips for single-cell AST are fabricated using well-established soft lithography with PDMS and glass, which is amenable to standardization and scaling. These approaches avoid the high costs of silicon-based cleanroom fabrication, significantly lowering the per-device cost and enabling disposable use-a prerequisite for point-of-care testing to prevent cross-contamination.

Further cost reduction and simplification are achieved through innovative designs that eliminate reliance on bulky external instruments. The self-priming DMF chip incorporates microvalves that automate fluid partitioning, removing the need for expensive external pumps or vacuum systems, thereby reducing both the complexity and cost of the overall testing platform (Pang et al., 2025). Even greater affordability is realized with paper-based or paper/PDMS hybrid microfluidics. One developed hybrid chip leverages the capillary force of paper to drive flow and uses PDMS to form sample reservoirs, creating a completely pump-free system. The material and manufacturing costs for such a chip are reported to be under $1, making it exceptionally suitable for widespread screening in resource-limited settings (Zhu et al., 2024). This design philosophy prioritizes “frugal science, “ aiming to maximize functionality while minimizing material and operational expenses.

The economic viability is also enhanced by integrating multiple diagnostic functions into a monolithic, ready-to-use device. Advanced platforms, such as the SDA chip, consolidate pathogen quantitative detection, identification, and phenotypic AST into a streamlined workflow (Diao et al., 2025). This integration drastically reduces the total turnaround time compared to sequential traditional methods. By pre-storing reagents on-chip and automating the entire process from sample to answer, these integrated systems minimize hands-on time, reduce the need for skilled technicians, and lower the overall operational cost per test. Consequently, the manufacturing economics of these next-generation microfluidic chips are evaluated not merely on unit cost, but on their total value proposition in enabling rapid, precise, and accessible AMR diagnostics.

Further cost reduction and simplification are achieved through innovative designs that eliminate reliance on bulky external instruments. The self-priming DMF chip incorporates microvalves that automate fluid partitioning, removing the need for expensive external pumps or vacuum systems, thereby reducing both the complexity and cost of the overall testing platform (Pang et al., 2025). Even greater affordability is realized with paper-based or paper/PDMS hybrid microfluidics. One developed hybrid chip leverages the capillary force of paper to drive flow and uses PDMS to form sample reservoirs, creating a completely pump-free system. The material and manufacturing costs for such a chip are reported to be under $1, making it exceptionally suitable for widespread screening in resource-limited settings. This design philosophy prioritizes “frugal science, “ aiming to maximize functionality while minimizing material and operational expenses (Nge et al., 2013). The economic viability is also enhanced by integrating multiple diagnostic functions into a monolithic, ready-to-use device. Advanced platforms, such as the SDA chip, consolidate pathogen quantitative detection, identification, and phenotypic AST into a streamlined workflow (Diao et al., 2025). This integration drastically reduces the total turnaround time compared to sequential traditional methods. By pre-storing reagents on-chip and automating the entire process from sample to answer, these integrated systems minimize hands-on time, reduce the need for skilled technicians, and lower the overall operational cost per test. Consequently, the manufacturing economics of these next-generation microfluidic chips are evaluated not merely on unit cost, but on their total value proposition in enabling rapid, precise, and accessible AMR diagnostics. **Table 5** clearly compares the four microfluidic technology pathways used for rapid detection of antibiotic-resistant bacteria, focusing on their core manufacturing strategies and economic characteristics.

**Table 5:** Economic characteristics and key limitations of representative microfluidic technology pathways.

Technology platform/Name	Core manufacturing materials & Processes	Economic strategy & Key advantages	Key limitations	Target application scenario	References
Centrifugal Microfluidic Chip System	Polymers (e.g., Cyclic Olefin Copolymer, COC); Injection Molding	Reduces per-unit cost through mass production via injection molding; integrated design shortens the multi-day workflow to within 48 hours.	Still requires a dedicated spinning/reading instrument; cartridge design and fluidic balancing can increase engineering complexity; less suitable for extremely low-resource settings without supporting hardware	Suitable for hospital laboratories, enabling rapid resistance assessment and antibiotic selection.	(Xu et al., 2024)
Self-Priming Digital Microfluidic (DMF) Chip	PDMS, Glass; Soft Lithography	Utilizes mature, low-cost soft lithography; chip-integrated microvalves eliminate the need for expensive external pumps, reducing total system cost.	PDMS/glass fabrication may be less scalable than thermoplastic mass production; device operation and signal readout can still require specialized control electronics; throughput may remain limited for routine large-scale screening	Ideal for point-of-care testing, achieving antibiotic susceptibility testing (AST) at the single-cell level within 6 hours.	(Pang et al., 2025)
Paper/PDMS Hybrid Microfluidic Chip	Filter Paper, PDMS; Lamination	Leverages capillary action of paper for pump-free operation; material and manufacturing cost is under $1.	Limited multiplexing and quantitative precision compared with more integrated chip platforms; paper-based formats may be less robust for complex clinical matrices and long multi-step workflows	Designed for primary screening, and on-site detection in environmental or food safety monitoring, prioritizing ultra-low per-test cost.	(Zhu et al., 2024)
Static Droplet Array (SDA) Chip	Polymers; Microfabrication	High integration (quantification + identification+AST); the monolithic “sample-in-answer-out” design reduces manual operation and total turnaround time (~12 hours), lowering overall costs.	Higher system integration may increase fabrication complexity and instrument dependence; implementation may require advanced imaging, identification, or data-analysis support; total platform cost may remain higher than that of simpler screening-oriented devices	Targets critical infections like bloodstream infections, providing a complete etiological diagnosis within the critical “golden window”.	(Diao et al., 2025)

### 7.2 Regulatory hurdles and standardization gaps

Despite the remarkable technical advances in microfluidic platforms for rapid AST, their transition from laboratory prototypes to clinically integrated tools is significantly hampered by substantial regulatory hurdles and a lack of standardization. A primary barrier is the absence of universally accepted performance standards and validation protocols specifically tailored for these novel microfluidic diagnostic systems (Jack et al., 2024). Current regulatory frameworks, such as those enforced by the U.S. FDA or the European Medicines Agency (EMA), are primarily designed for traditional, well-established diagnostic methods. They often lack clear guidance for evaluating the unique aspects of microfluidic devices, which may combine rapid phenotypic growth monitoring, genotypic detection, and novel signal transduction methods into a single, miniaturized platform. This creates uncertainty for developers regarding the necessary clinical trial design, comparator methods, and the acceptable thresholds for sensitivity, specificity, and limit of detection required for regulatory approval.

The WHO’s consultation on AMR diagnostic standards highlighted critical gaps: no consensus exists on acceptable limits of detection for low-abundance resistance genes in blood, nor on criteria for distinguishing colonization from infection in multiplex panels.The path to market clearance is further complicated by the need to demonstrate analytical robustness and clinical utility across diverse settings. Devices intended for point-of-care use must prove they can deliver reliable results when operated by non-specialist personnel in variable environments, not just in controlled laboratory conditions. Additionally, achieving consensus on standardized materials, fabrication quality control, and data interpretation algorithms is critical for ensuring that results are reproducible and comparable across different devices and laboratories (Song et al., 2022). Without such standardization, even approved technologies may face slow and fragmented clinical adoption.

Bridging this translational chasm requires a concerted, multi-stakeholder effort. Regulatory science must evolve in tandem with diagnostic innovation. Agencies and expert bodies need to develop flexible, fit-for-purpose evaluation frameworks that recognize the unique value proposition of rapid, integrated AST while safeguarding clinical safety and efficacy. This could involve creating new device classifications or endorsing modular approval approaches for validated technological components. Concurrently, there is an urgent need for international consensus-building on technical standards.Only through such proactive collaboration can the full potential of microfluidic AMR diagnostics be realized, transforming them from impressive prototypes into reliable, accessible tools that are routinely used to guide therapy and combat the global antimicrobial resistance crisis.

### 7.3 Workflow integration

The ultimate value of microfluidic rapid AST platforms is determined not only by their analytical performance in the laboratory but also by their seamless and effective integration into real-world clinical workflows. The operational demands and resource landscapes vary drastically between settings such as ICU and community or primary care clinics. Successful translation, therefore, hinges on strategic platform design that prioritizes either extreme speed and comprehensiveness for critical care or maximal simplicity, robustness, and low cost for decentralized settings.

In the ICU, where patients with suspected bloodstream infections or severe pneumonia are critically ill, the primary driver is time-to-action. Each hour of delay in administering effective antimicrobial therapy is associated with a significant increase in mortality. Here, the workflow integration challenge centers on delivering a definitive pathogen identification and a reliable phenotypic AST profile within a single dosing interval of broad-spectrum empiric antibiotics (Pratt et al., 2025). The goal is to enable de-escalation or targeted escalation before the second antibiotic dose is due. This requires platforms that can directly handle complex, primary samples (like whole blood or respiratory specimens), minimize hands-on time for busy clinical staff, and integrate results directly into the hospital’s electronic medical record for immediate clinical decision-making.

In contrast, community clinics, doctor’s offices, or resource-limited settings face a different set of constraints. The priorities shift towards accessibility, ease-of-use, low per-test cost, and minimal infrastructure dependency. Operators may have limited technical training, and there is no access to central laboratory equipment or support. Therefore, the ideal platform for these environments must be truly “sample-in, answer-out, “ with minimal processing steps, battery-operated or low-power instrumentation, and intuitive result readouts (Zai et al., 2025). The workflow integration focuses on enabling testing at the first point of patient contact, avoiding sample referral delays, and guiding initial therapeutic decisions without relying on distant laboratories.

To meet these divergent needs, recent microfluidic platforms have evolved along distinct but complementary design philosophies. For ICU and hospital laboratory integration, the focus is on speed, automation, and processing complex samples. The “nano-dilution SlipChip” (nd-SlipChip) exemplifies this by enabling phenotypic AST and even assessment of antibiotic combinations or phage susceptibility within 2–4 hours from a positive culture, using a simple manual sliding mechanism to create nano-droplet assays (Wang et al., 2025). For more severe cases requiring direct-from-blood analysis, platforms like the SDA chip system integrate pathogen quantitative detection, MALDI-TOF identification, and AI-driven MIC analysis in approximately 12 hours directly from untreated whole blood, offering a dramatic acceleration over the standard 48–72 hour culture-based pipeline. Similarly, platforms like Pattern Bioscience’s technology use single-cell microfluidics and AI to provide both pathogen ID and AST results in about 4 hours from clinical specimens, specifically targeting pneumonia and bacteremia in hospitalized patients. Another strategy uses specialized microfluidic chips for high-throughput Dean Flow Fractionation (DFFHT) to rapidly separate bacteria from blood cells, coupling this with specific aptamer-based detection of resistance markers to deliver a diagnostic result in under 1 hour post-culture positivity (Wu et al., 2026).

For community and point-of-care integration, the design paradigm emphasizes extreme simplification, low cost, and connectivity. Successful platforms often leverage isothermal amplification (like RPA or LAMP) to eliminate the need for costly thermal cyclers (Suarez et al., 2025). A prime example is a handheld, battery-operated molecular POCT system designed for home use. It uses a disposable microfluidic cartridge for multiplexed respiratory pathogen detection, features one-button operation, and wirelessly transmits results to a smartphone, with a per-test cost targeted below $1.4 (Zai et al., 2025). Another approach employs injection-molded centrifugal microfluidic chips with integrated optical sensors. This mass-producible design, with a chip cost around $5, automates RT-LAMP assays for virus detection in under 50 minutes, requiring only a simple spinning and reading device. The ultimate in simplicity is demonstrated by platforms like the portable Tip-Optical-fluidic Immunoassay (TOI), which uses a 1 µL finger-prick blood sample in a disposable microfluidic tip that attaches to a standard pipettor, delivering quantitative serology results in 40 minutes (Tan et al., 2025).

Beyond device-specific features, broader workflow integration is facilitated by designing for connectivity and scalability. The future lies in systems that not only generate a rapid result but also electronically report it, facilitating antimicrobial stewardship by alerting pharmacists and clinicians, and enabling real-time, anonymized surveillance of local and regional AMR trends. Furthermore, manufacturing strategies like injection molding are critical for transitioning from lab prototypes to the consistent, high-volume production required for widespread clinical deploymen.

## 8 Future trajectories toward intelligent, multiplexed antimicrobial susceptibility testing

### 8.1 Next-generation cartridge

The evolution of AST is shifting from sequential, culture-dependent workflows toward integrated, sample-to-answer platforms capable of delivering comprehensive diagnostic intelligence within a single microfluidic cartridge. Next-generation cartridges now combine nucleic acid amplification, CRISPR-based detection, and phenotypic response monitoring to concurrently identify pathogens, map resistance determinants, and predict therapeutic efficacy—transforming AST from a passive reporting tool into an active clinical decision engine. Recent prototypes leverage multiplexed loop-mediated isothermal amplification (mLAMP) panels targeting over 50 clinically relevant resistance genes (*blaKPC, mecA, vanA*, etc.) alongside species-specific 16S rRNA markers, achieving pathogen identification and genotypic resistance profiling in under 45 minutes with >98% concordance against whole-genome sequencing benchmarks (Kaur et al., 2024). Crucially, these systems embed interpretive algorithms that translate genetic findings into actionable therapy recommendations aligned with local antibiograms and CLSI/EUCAST guidelines, thereby reducing cognitive burden on clinicians and minimizing guideline-discordant prescribing.

Phenotypic validation remains indispensable, as genotypic predictions may miss novel or uncharacterized resistance mechanisms. To address this, advanced cartridges integrate real-time metabolic monitoring using redox-sensitive dyes or impedance-based growth curves within nanoliter-scale culture chambers. For instance, a platform developed by Sathish et al. employed parallel microchambers containing gradient antibiotic concentrations, with bacterial growth tracked via time-lapse fluorescence imaging; machine learning classifiers then inferred minimum inhibitory concentrations (MICs) from early kinetic signatures (Sathish and Shen, 2021). This hybrid genotypic–phenotypic approach mitigates the limitations of either modality alone: genotyping provides speed and breadth, while phenotyping captures functional resistance, including heteroresistance and adaptive tolerance.

Cartridge architecture has also evolved to support complex fluidic operations without external pumps. Centrifugal microfluidics, for example, uses rotational force to sequentially release reagents from blister packs into reaction zones, enabling multi-step protocols on a disposable disc (García-Azuma et al., 2024). Similarly, capillary-driven paper-polymer hybrids exploit wicking forces to move samples through stacked layers containing dried reagents, achieving fully autonomous operation ideal for low-resource settings (Chai et al., 2021; Hamidon et al., 2021). These designs significantly reduce user intervention, a critical factor for point-of-care deployment where technician expertise is limited.Performance metrics across emerging platforms demonstrate substantial gains in sensitivity and turnaround time compared to conventional methods(**Table 6**).

**Table 6:** Comparative performance characteristics of representative integrated microfluidic platforms for rapid AMR detection and susceptibility profiling.

Platform type	Pathogen/target identification time	Resistance marker panel size	Phenotypic AST/MIC time	Limit of detection (CFU/mL)	Sample type	References
CRISPR-microfluidic platform	35 min	48 genes	Not included	10²	Blood, urine	(Kaur et al., 2024)
mLAMP-impedance platform	40 min	32 genes	90 min	10³	Positive blood culture	(Sathish and Shen, 2021)
Paper-based lateral flow assay (LFA) platform	25 min	8 genes	Not included	10^4^	Urine	(García-Azuma et al., 2024)
Centrifugal microfluidic AST platform	50 min	20 genes	120 min	10³	Whole blood	(Hamidon et al., 2021)

Despite these advances, challenges persist in multiplex assay design. Cross-reactivity between primer sets can cause false positives, while matrix effects from complex clinical specimens (e.g., hemoglobin in blood, mucins in sputum) may inhibit enzymatic reactions. Strategies to mitigate these include on-chip sample purification using inertial focusing to isolate bacteria from plasma, or incorporating internal amplification controls to flag inhibition events (Kaur et al., 2024). Furthermore, regulatory pathways must adapt to validate such multifunctional devices; current FDA frameworks evaluate each intended use claim separately, potentially requiring redundant clinical trials for what is functionally a unified diagnostic system. As resistance landscapes grow more complex—particularly with pan-resistant *Acinetobacter* and *Candida auris*—the ability to rapidly de-escalate or switch to last-resort agents becomes not just clinically advantageous but ethically imperative.

### 8.2 AI-integrated microfluidics

The integration of artificial intelligence (AI) with microfluidic AST platforms marks a paradigm shift from static, predetermined testing protocols to dynamic, feedback-driven diagnostic strategies that optimize resource use and accelerate result generation. Traditional AST follows fixed incubation timelines, irrespective of bacterial growth kinetics or early response signals. In contrast, AI-enhanced microfluidics continuously analyze real-time data streams—optical density, fluorescence intensity, impedance shifts—to detect subtle phenotypic changes and adapt testing parameters on-the-fly. Convolutional neural networks (CNNs) trained on time-lapse microscopy of bacterial microcolonies can predict final MICs with >90% accuracy after only 3–4 hours of growth by recognizing morphological patterns associated with resistance, such as filamentation in β-lactam-exposed *E. coli* or suppressed division in vancomycin-treated *S. aureus* (Jian et al., 2024).

AI integration is pivotal for deciphering complex, multiplexed signals to achieve ultra-rapid, combined identification and AST (Monaco et al., 2025). One sophisticated system combines a microfluidic valve-based platform for combinatorial antibiotic assembly, a laser-induced fluorescent (LIF) detection system at single-cell resolution, and a machine learning algorithm for identification. This system utilizes an innovative assay that hybridizes fluorescent oligonucleotide probes with bacterial 16S ribosomal RNA. A multidimensional Quadratic Discriminant Analysis (QDA) machine learning model then processes the resulting complex signal patterns, enabling automated identification of seven different bacterial species with 98% accuracy in under 5 minutes. Furthermore, the platform performs high-throughput combinatorial AST in under 1 hour (Wei et al., 2025). In another paradigm, surface-enhanced Raman spectroscopy (SERS) signals acquired directly within a microfluidic chip are transformed into 2D representations. Optimized 2D-CNNs trained on these representations have achieved up to 99% accuracy for bacterial classification on controlled datasets and demonstrated perfect adaptability (100% accuracy) to new on-chip data via transfer learning, showcasing a powerful tool for low-volume, rare infection diagnostics (Feizpour et al., 2025).

Beyond classification, AI algorithms enhance the quantification of viable bacteria and the monitoring of subtle growth kinetics. A digital microfluidic chip designed for multiplex detection uses a custom deep learning model (TLENTNet) to analyze time-lapse images of bacteria growing in an array of micro-wells. The model recognizes colonies based on spatiotemporal growth features, automatically counts positive wells, and quantifies bacterial concentration, achieving results highly consistent with the gold standard plate count method but in a fraction of the time (Quan et al., 2023). To overcome sensitivity limits in monitoring metabolic activity, platforms like the Synchronized droplet-amplified electrical screening cytometry (SYNC) system have been developed. SYNC encapsulates single bacteria in droplets and uses a phosphorylation-amplified culture medium to translate metabolic activity into measurable impedance changes. While this study highlights a 5-fold enhancement in sensitivity and a 50% reduction in detection time compared to traditional impedance cytometry, the vast amount of real-time impedance kinetic data generated is inherently suited for further analysis by AI algorithms to predict antibiotic susceptibility earlier and more accurately (Zhong et al., 2024). The application of machine learning for such continuous, real-time signal analysis is recognized as a key direction for maximizing the utility of complex microfluidic datasets (Asadian et al., 2024).

Despite the transformative potential, several challenges remain for the widespread clinical adoption of AI-integrated microfluidics. A significant hurdle is the “black box” nature of many complex deep learning models, which can hinder clinical trust and regulatory approval. Developing explainable AI frameworks that provide insights into the decision-making process of these models is crucial. Furthermore, the performance of AI models is heavily dependent on the quality and diversity of their training data. There is a pressing need for large, standardized, and clinically relevant datasets that encompass a wide range of bacterial strains, antibiotic concentrations, and sample matrices to ensure robustness and generalizability (Hallström et al., 2025). Future research must focus on creating unified, adaptive AI architectures that can be efficiently trained or fine-tuned with limited clinical data from a specific setting. Ultimately, the goal is to evolve these systems into fully autonomous diagnostic platforms where AI not only interprets data but also dynamically adjusts testing parameters—such as imaging intervals or antibiotic concentration gradients—in real-time based on initial bacterial response, paving the way for truly personalized and rapid antimicrobial therapy.

### 8.3 Building a global diagnostic ecosystem

Realizing the full potential of next-generation AST technologies demands more than technical innovation—it requires a coordinated global ecosystem that aligns developers, regulators, clinicians, and public health stakeholders around shared standards, equitable access, and context-appropriate design. Historically, diagnostic development has followed a linear “bench-to-bedside” model optimized for high-income markets, resulting in sophisticated tools ill-suited for the infrastructure, disease burdens, and economic realities of LMICs, where AMR mortality is highest. A new co-development paradigm, grounded in interdisciplinary collaboration from inception, is essential to ensure that intelligent microfluidic platforms deliver impact across the entire care continuum.

Central to this ecosystem is the principle of “frugal innovation”—designing for performance, durability, and affordability without compromising clinical utility. Equally important are regulatory harmonization mechanisms such as the African Union’s African Medicines Agency, which aims to streamline approval across 55 nations, reducing redundant trials and accelerating access. Validation must extend beyond analytical performance to real-world clinical utility. Traditional diagnostic studies focus on sensitivity/specificity against reference methods, but for AST, the gold standard—broth microdilution—is itself imperfect and slow. New endpoints are needed, such as time-to-appropriate-therapy, antimicrobial consumption reduction, or mortality benefit.

Crucially, this ecosystem must prioritize equity. Technologies should be designed with input from end-users in diverse settings—from ICU intensivists to rural community health workers—to avoid “solutionism” that ignores workflow realities. Training programs and digital support tools (e.g., AR-guided cartridge loading) can bridge skill gaps, while telemedicine networks enable remote expert consultation for complex cases. Ultimately, the goal is not merely to deploy more diagnostics but to embed them within strengthened health systems where every test result translates into timely, life-saving action against the rising tide of antimicrobial resistance.

## 9 Conclusion

The rapid detection of antibiotic-resistant bacteria stands at the forefront of global efforts to combat the escalating AMR crisis, and microfluidic chip technology has emerged as a transformative force in this endeavor. By miniaturizing and integrating complex laboratory processes onto disposable, often automated platforms, microfluidics bridges critical gaps left by conventional culture-based diagnostics—dramatically reducing turnaround times from days to under an hour while preserving or even enhancing analytical sensitivity and specificity. The convergence of phenotypic and genotypic modalities within single devices enables unprecedented insights into bacterial behavior, capturing not only known resistance genes but also functional responses, heteroresistance, and adaptive tolerance that dictate clinical outcomes. Innovations such as CRISPR-Cas integration, nanomaterial-enhanced biosensing, and AI-driven data interpretation have further elevated these platforms from simple detectors to intelligent diagnostic ecosystems capable of guiding precision antimicrobial therapy in real time. Validation studies across diverse clinical matrices—including blood, urine, and sputum—confirm robust performance even in complex, low-burden specimens, with several systems now demonstrating mortality benefits and stewardship gains in prospective trials. Nevertheless, the path from technological promise to global health impact remains fraught with translational challenges: manufacturing economics must balance affordability with functionality across high- and low-resource settings; regulatory frameworks require harmonization to avoid redundant validation; and seamless workflow integration demands context-aware design co-developed with end-users. Looking ahead, next-generation cartridges that unify pathogen identification, resistance mechanism mapping, and therapeutic recommendation in a single assay—powered by adaptive AI algorithms and deployed within equitable, interdisciplinary diagnostic ecosystems—hold the potential to redefine antimicrobial stewardship worldwide. Realizing this vision will necessitate sustained collaboration among engineers, clinicians, microbiologists, public health experts, and policymakers to ensure that these powerful tools are not only scientifically advanced but also accessible, actionable, and aligned with the urgent needs of patients and populations across the global care continuum.
